# NADH/NADPH bi-cofactor-utilizing and thermoactive ketol-acid reductoisomerase from *Sulfolobus acidocaldarius*

**DOI:** 10.1038/s41598-018-25361-4

**Published:** 2018-05-08

**Authors:** Chin-Yu Chen, Tzu-Ping Ko, Kuan-Fu Lin, Bo-Lin Lin, Chun-Hsiang Huang, Cheng-Hung Chiang, Jia-Cherng Horng

**Affiliations:** 1 Department of Life Sciences, National Central University, Taoyuan, 32001 Taiwan; 2 Institute of Biological Chemistry, Academia Sinica, Taipei, 11529 Taiwan; 3 Research Center for Applied Sciences, Academia Sinica, Taipei, 11529 Taiwan; 4 Protein Diffraction Group, Experimental Facility Division, National Synchrotron Radiation Research Center, Hsinchu, 30076 Taiwan; 5 Department of Chemistry, National Tsing Hua University, Hsinchu, 30013 Taiwan

## Abstract

Ketol-acid reductoisomerase (KARI) is a bifunctional enzyme in the second step of branched-chain amino acids biosynthetic pathway. Most KARIs prefer NADPH as a cofactor. However, KARI with a preference for NADH is desirable in industrial applications including anaerobic fermentation for the production of branched-chain amino acids or biofuels. Here, we characterize a thermoacidophilic archaeal Sac-KARI from *Sulfolobus acidocaldarius* and present its crystal structure at a 1.75-Å resolution. By comparison with other holo-KARI structures, one sulphate ion is observed in each binding site for the 2′-phosphate of NADPH, implicating its NADPH preference. Sac-KARI has very high affinity for NADPH and NADH, with *K*_*M*_ values of 0.4 μM for NADPH and 6.0 μM for NADH, suggesting that both are good cofactors at low concentrations although NADPH is favoured over NADH. Furthermore, Sac-KARI can catalyze 2(*S*)-acetolactate (2*S*-AL) with either cofactor from 25 to 60 °C, but the enzyme has higher activity by using NADPH. In addition, the catalytic activity of Sac-KARI increases significantly with elevated temperatures and reaches an optimum at 60 °C. Bi-cofactor utilization and the thermoactivity of Sac-KARI make it a potential candidate for use in metabolic engineering or industrial applications under anaerobic or harsh conditions.

## Introduction

**Figure 1:**
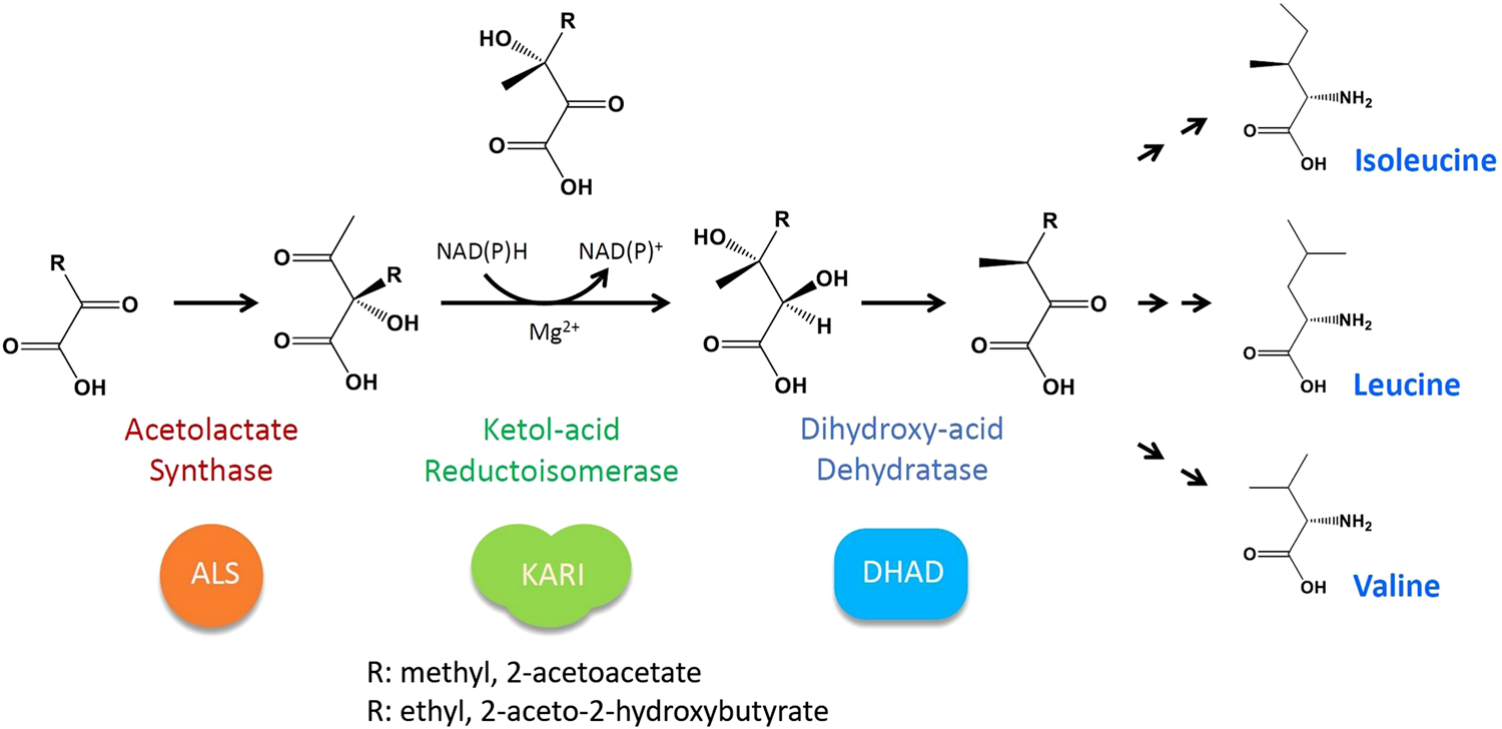
Biosynthetic pathways for BCAA. In the first reaction acetolactate synthase (ALS) condenses one pyruvate with another into 2-acetoacetate (for valine and leucine) or with 2-ketobutyrate to form 2-aceto-2-hydroxybutyrate (for isoleucine). Both products are then isomerized and reduced *via* keto-acid reductoisomerase (KARI) to form 2,3-dihydroxyisovalerate and 2,3-dihydroxy-3-methylvalerate, respectively. In this unusual two-step reaction, each substrate undergoes Mg^2+^-dependent alkyl migration followed by NAD(P)H-dependent reduction of the 2-keto group. Dihydroxyacid dehydratase (DHAD) converts two products of the second step to 2-ketoisovalerate and 2-keto-3-methylvalerate, respectively. The final step in the parallel pathway is conducted catalysed by amino transferase, which yields the final products of valine from 2-ketoisovalerate and isoleucine from 2-keto-3-methylvalerate. In contrast, a series of more additional enzymes are necessary to sequentially divert 2-ketoisovalerate to the leucine production pathway.

Isoleucine, leucine and valine are essential in the diets of animals because animals lack the machinery for producing these branched-chain amino acids (BCAAs). The BCAAs are synthesized from pyruvate by a common pathway that involves acetolactate synthase (ALS), ketol-acid reductoisomerase (KARI), and dihydroxy-acid dehydratase (DHAD). As a bifunctional key enzyme, KARI catalyzes an essential two-step reaction for BCAA biosynthesis (Fig. 1). KARI is specific for the (*S*) enantiomer of both 2-acetolactate (2-AL) and 2-aceto-2-hydroxy-butyrate (AHB) in the biosynthesis of valine (2AL) and isoleucine (AHB)^1–3^. The reaction comprises Mg^2+^-dependent alkyl shift and carbonyl group reduction by NADPH^4,5^ or NADH^6,7^. Previous enzyme kinetic studies have shown that the isomerase and reductase activity of KARI can be measured separately *in vitro* by using natural or artificial substrates^8,9^, and the reductase activity is capable of utilizing a variety of 2-keto acids^8^. Because animals lack this enzyme, KARI is a target enzyme for the development of herbicides and antibiotics^10–13^. In addition to the production of essential amino acids for food and feed^14,15^, the BCAA pathway has also been manipulated to exploit microorganisms for manufacturing biofuels^16,17^.

There are two classes of KARIs. Class I KARIs are shorter in polypeptide chain length (~340 aa), and class II KARIs are longer (~490 aa). Proteins of both classes fold into an N-terminal Rossmann domain and a C-terminal “knot” domain. In the Class I enzymes, two knot domains of two monomers intertwine to form a homodimer^7,13^. In *Pseudomonas aeruginosa* and *Helicobacter pylori*, the dimers further associate into dodecamers^18^. In Class II KARIs, these enzymes contain duplicated knot domains to form the active site within a single subunit. They exist in a dimer form in plants or as a tetramer in *Escherichia* *coli* KARI^19^. The active site in both forms is located between the N- and C-terminal domains, and the β2-αB loop of the N-terminal domain determines the enzyme’s specificity for NADPH or NADH. Because living cells usually contain higher levels of NADH but most KARIs prefer NADPH as the reductive cofactor, attempts have been made to engineer KARIs for more efficient substrate conversion using NADH^17,20,21^.

Anaerobic conditions are more economically feasible for large-scale production than aerobic conditions. Under anaerobic conditions, however, the only available reducing cofactor is the NADH generated through glycolysis. Therefore, KARIs with preference for the NADH cofactor are desirable for industrial applications, including anaerobic fermentation for the production of branched-chain amino acids or biofuels. Based on multiple sequence alignment, information from available holo-formed structures^7,13,19,20,22–26^, and engineering studies of KARIs^17,20,21^, Brinkmann-Chen *et al*. showed that the acidic residues at conserved NADPH phosphate-binding positions in the β2-αB loop can be used to identify putative NADH-preferring KARIs in nature. Prior to the characterization of several native NADH-dependent KARIs^7,8,27^, this family of enzymes was believed to be exclusively NADPH-dependent.

Resistance to harsh conditions is another important consideration when choosing an enzyme for industrial applications. In this study, we present the crystal structure of a class-I KARI from the thermoacidophilic archaeon *Sulfolobus acidocaldarius* at 1.75-Å resolution. This enzyme, which we refer to here as Sac-KARI, was predicted to prefer NADPH based on the structure of its cofactor binding site. According to the sequence classification and cofactor specificity prediction performed by Brinkmann-Chen *et al*., Sac-KARI has a loop sequence (LEREGNS) that suggests a putative bispecific cofactor binding mode similar to that found in KARIs from *Ignisphaera aggregans* (Ia-KARI) and *Metallosphaera sedula* (Ms-KARI)^6^. Here, in addition to analysing the thermostability and acid tolerance of Sac-KARI by circular dichroism (CD) spectroscopy, we further investigate the temperature dependence of Sac-KARI activity and confirm its cofactor preference.

## Results

### Sac-KARI forms a heat-stable homodimer

Recombinant Sac-KARI was overexpressed in *Escherichia coli* BL21 (DE3) and purified by heating at 65 °C for 30 minutes followed by nickel-NTA affinity chromatography and gel filtration chromatography. Gel filtration showed that in the presence of 2 mM Mg^2+^ Sac-KARI is a dimer with a molecular weight of ~70 kDa (the theoretical molecular weight of the dimer is ~74 kDa). This contrasts with KARI from *Pseudomonas aeruginosa* (Pa-KARI)^18^, which forms a dodecamer, and *Escherichia coli* KARI (Ec-KARI)^19^, which forms a tetramer, but is similar to the spinach (So-KARI)^22^, *Slackia exigua* (Se-KARI)^20^ and *Mycobacterium tuberculosis* (Mt-KARI)^13^ enzymes, which are dimers.

**Figure 2:**
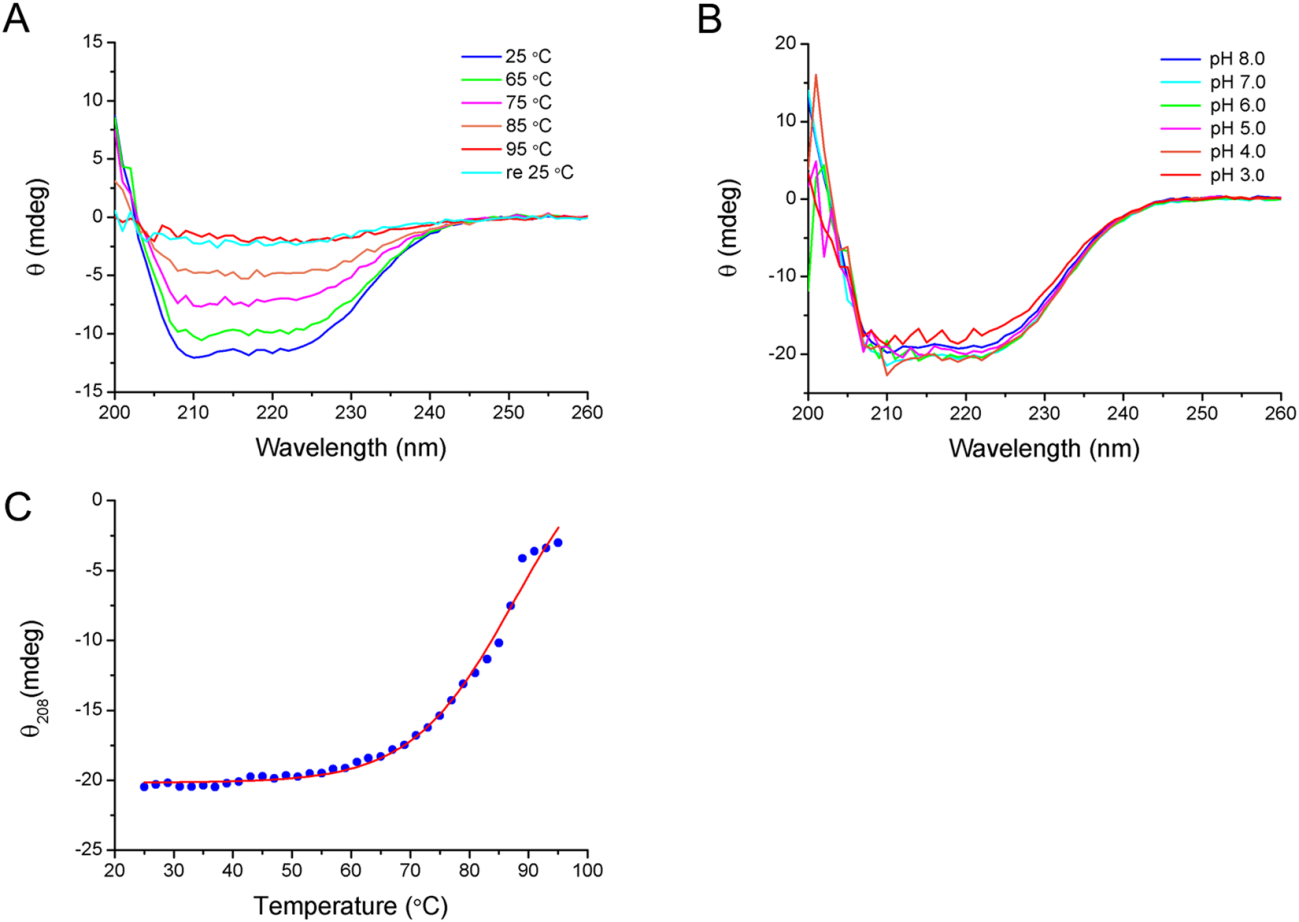
CD spectra of Sac-KARI in phosphate buffer (pH 8.0) containing 2 mM Mg^2+^. (**A**) The far-UV CD spectra of Sac-KARI measured over the wavelength range 200 to 260 nm at temperatures from 25 to 95 °C. (**B**) The far-UV CD spectra show that the secondary structure of Sac-KARI is unaffected by changes in pH over the range of pH values from 3.0 to 8.0. (**C**) CD-monitored thermal unfolding curve of Sac-KARI at 208 nm. Fitting of the thermal melting curve indicates that the *Tm* of Sac-KARI is approximately 86 °C.

Archaeal enzymes from extremophiles often display tolerance to adverse conditions under which a mesophilic counterpart usually loses its activity due to unfolding of the polypeptide chain. By comparing the CD spectra of Sac-KARI at various temperatures (Fig. 2A), it is clear that the enzyme retained most of its native folding, when the temperature was increased to 65 °C. At 95 °C, the enzyme was completely unfolded, and it did not refold even when it was slowly cooled to 25 °C. Presumably the unfolding process is irreversible, especially after dissociation of the intertwined knot domains. On the other hand, variations in pH from 3 to 8 did not significantly alter the CD spectra (Fig. 2B), as expected for an enzyme from an acidophilic organism. Sac-KARI exhibited pH tolerance over a broader range (pH 3–8), which is preferred in industrial application.

The thermal stability of a protein can be measured quantitatively, and the melting temperature at which 50% of the protein molecules are unfolded (*Tm*) during the thermal unfolding transition can be determined. The thermal stability of Sac-KARI was assessed using CD by following changes in the CD spectrum with increasing temperature at a wavelength of 208 nm (Fig. 2C). Although the thermal melting curve fitted to the data indicates that the *Tm* of Sac-KARI is ~86 °C, the curve also suggests that the protein may begin to denature at temperatures above 60 °C. Consequently, we chose 60 °C as the elevated temperature for measurement of the enzyme activity.

### Structural analysis of Sac-KARI suggests a preference for NADPH

**Figure 3:**
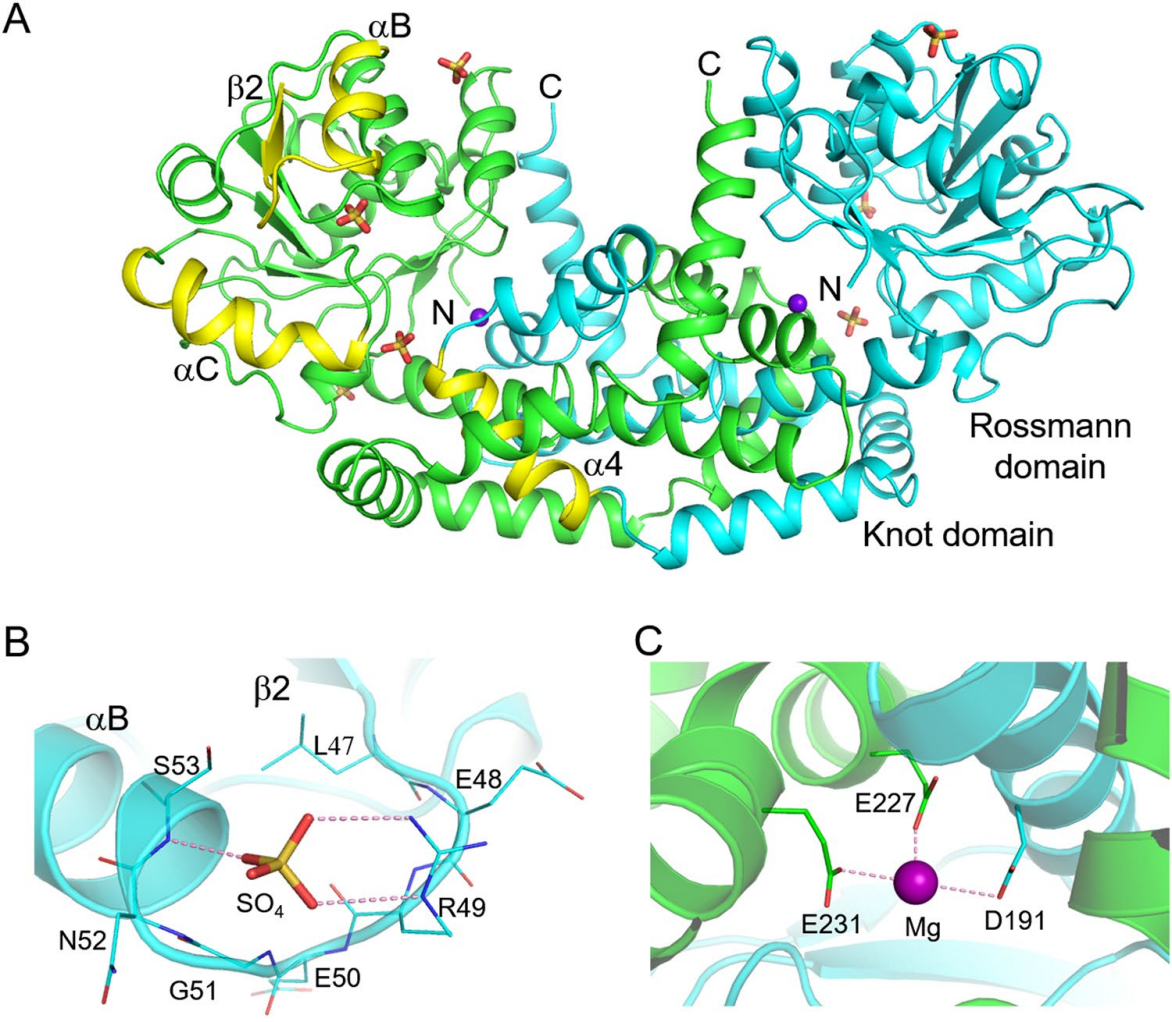
Crystal structure of Sac-KARI. (**A**) A dimer comprising the asymmetric unit is shown as a ribbon model with the monomers coloured green and cyan. The N and C termini are indicated, as are the Rossmann and knot domains and a few secondary structural elements of interest, which are coloured yellow. The bound sulphate and magnesium ions are shown as stick-and-sphere models. (**B**) The second sulphate interacts with the β2-αB loop. It can form hydrogen bonds with R49 and S53, as does the 2′-phosphate of NADPH. (**C**) Magnesium binding. The bound magnesium ion is shown as a purple sphere. The surrounding acidic side chains are shown as stick models. D191 is from the second monomer, which is coloured cyan rather than green.

The crystal structure of Sac-KARI was refined to 1.75-Å resolution, yielding low R values and good geometry. Some refinement statistics are given in Table 1. Each monomer of Sac-KARI folds into an N-terminal Rossmann domain and a C-terminal “knot” domain (Fig. 3A). The latter intertwines with the knot domain of another monomer to form a dimer similar to the MR search model of *Ignisphaera aggregans* KARI (Ia-KARI, PDB ID 4XDZ). The strands and helices in the N-terminal domain are designated β1 to β8 and αA to αG, respectively, and the helices in the C-terminal domain are designated α1 to α7 (Supplemental Fig. S1). A comparison with holo-Ia-KARI, Mt-KARI-Mg^2+^ and Sac-KARI structures bound to different ligands suggests that both Sac-KARI and Mt-KARI are in an open conformation in which the N-terminal domain is rotated away from the C-terminal domain, exposing the active site to solvent (Supplemental Fig. S2). The disposition of N-terminal domain relative to C-terminal domain in Mt-KARI is more open than that in Sac-KARI. In contrast, the N-terminal domain of Ia-KARI is pulled closer to the C-terminal domain due to the binding of NADPH and IpOHA. The open-close transition has been thought to facilitate substrate binding and catalysis.

**Table 1:** Data collection and refinement statistics for Sac-KARI.

Data Collection	
Space group	*P*2_1_2_1_2_1_
Unit-cell *a*, *b*, *c* (Å)	48.4, 90.8, 154.1
Resolution (Å)^a^	30.0–1.75 (1.81–1.75)
No. of unique reflections	69,217 (6734)
Redundancy	4.4 (4.5)
Completeness (%)	99.4 (98.7)
Average I/σ(I)	33.8 (2.7)
Average CC_1/2_	0.946 (0.809)
R_merge_ (%)	3.6 (57.7)
**Refinement**	
Resolution (Å)	30.0–1.75 (1.81–1.75)
No. of reflections (work)	65,672 (4670)
No. of reflections (free)	3479 (240)
R_work_ (%)^b^	17.5 (25.2)
R_free_ (%)	21.6 (29.4)
Average B (Å^2^)/No. of atoms	
Protein	32.8/5144
Water	42.6/468
Ion	64.9/37
EDO	38.4/8
RMSZ/RMSD bond lengths (Å)	1.03/0.021
RMSZ/RMSD bond angles (°)	1.05/2.1
Ramachandran plot (%)	
Most favoured	96.5
Allowed	3.5
Clash score/Percentile^c^	3.45/98
Overall score/Percentile^c^	1.65/85
PDB ID code	5YEQ

^a^The values in parentheses show the statistics for the highest-resolution shells.

^b^R_work_ = (Σ_hkl_ ||F_o_| − |F_c_||)/Σ_hkl_ |F_o_|. R_free_ was calculated with 2.9% of the data excluded from refinement.

^c^Calculated by MolProbity.

The refined Sac-KARI model includes bound sulphate and magnesium ions and ethylene glycol. Three of the seven sulphate ions are bound nonspecifically to the surface of the protein. The other four are bound in pairs to identical sites on the two monomers. One sulphate is located near the N-terminus of helix αB. It undergoes virtually identical interactions with the 2′-phosphate of NADPH as those that occur in Ia-KARI^7^. These include two hydrogen bonds to the R49 side chain and one to the backbone NH of S53 (Fig. 3B). Together with the sequence comparison of the β2-αB loop LEREGNS with that of other KARIs (Supplemental Fig. S3 and Table S1), the bound sulphates in the cofactor binding site suggest that Sac-KARI prefers NADPH as a reducing cofactor. In addition, probably because of the presence of 2 mM magnesium in the lysis and purification buffers, a Mg^2+^ was seen in each active site, surrounded by the side chains of E227 and E231 of one monomer and D191 of another (Fig. 3C). The residues involved in metal ion-binding are conserved in class I KARIs. Mg^2+^ (I) is coordinated by D191 and E195 and Mg^2+^ (II) by D191, E227 and E231. D191 plays an important role by bridging both Mg^2+^. In Sac-KARI, only Mg^2+^ (II) is observed. Active-site comparison of class I KARIs reveals different Mg^2+^ locations and side chain conformations of D191, E227 and E231 in Sac-, Mt-, Se- and Ia-KARI bound to different ligands, but in the holo-structures of Ia-KARI and Se-KARI, they are highly similar (Supplemental Fig. S4). These results suggest that stringent active site and ligand interactions are necessary for an enzyme like KARI to carry out two-step reactions (alkyl migration and reduction).

### Sac-KARI can also use NADH for efficient catalysis

To further investigate the cofactor specificity of Sac-KARI, we measured its activity toward 2*S*-AL using NADPH or NADH as a cofactor in the presence of Mg^2+^ ions at 25, 37 and 60 °C (Table 2). The measured turnover numbers (*k*_*cat*_) for 2*S*-AL in the presence of NADPH ranged from 0.058 to 0.957 sec^−1^, and the *K*_*M*_ values were between 184 and 60 μM. In the presence of NADH, the *k*_*cat*_ for 2*S*-AL ranged from 0.055 to 0.526 sec^−1^, and the *K*_*M*_ values were between 469 and 91 μM. Thus, the use of NADPH results in higher substrate affinity (lower *K*_*M*_ values), higher turnover rate (*k*_*cat*_) and greater catalytic efficiency (*k*_*cat*_/*K*_*M*_) than the use of NADH. Nevertheless, despite its higher activity with NADPH than with NADH, these results show that Sac-KARI can use either NADPH or NADH as a cofactor in the reduction reaction.

**Table 2:** Kinetic parameters for the activity of KARI variants towards 2(*S*)-acetolactate in the presence of saturating concentrations of NADH or NADPH.

Enzyme (class)/cofactor	Origin	T (°C)	Mg^2+^ (mM)	*K*_*M*_ (μM)	*k*_*cat*_ (s^−1^)	*k*_*cat*_/*K*_*M*_ (mM^−1^ s^−1^)	Ref
Sac-KARI (I)/NADPH	*Solfolobus acidocaldarius*	25	2	184 ± 5	0.058 ± 0.001	0.32 ± 0.01	This work
			10	140 ± 6	0.068 ± 0.001	0.49 ± 0.02	
		37	2	157 ± 3	0.192 ± 0.000	1.23 ± 0.02	
			10	74 ± 5	0.195 ± 0.002	2.63 ± 0.14	
		60	2	93 ± 5	0.957 ± 0.003	10.30 ± 0.55	
			10	60 ± 4	0.876 ± 0.003	14.58 ± 0.92	
Sac-KARI (I)/NADH	*Solfolobus acidocaldarius*	25	2	469 ± 4	0.05 5 ± 0.001	0.12 ± 0.00	
			10	188 ± 5	0.06 2 ± 0.000	0.33 ± 0.01	
		37	2	165 ± 3	0.157 ± 0.001	0.95 ± 0.02	
			10	99 ± 2	0.164 ± 0.002	1.66 ± 0.03	
		60	2	144 ± 7	0.526 ± 0.013	3.66 ± 0.16	
			6	91 ± 4	0.444 ± 0.001	4.88 ± 0.22	
Mr-KARI (I)/NADPH	*Meiothermus ruber*	50	10	80 ± 10	0.77 ± 0.01	9.6 ± 1.8	^27^
Mr-KARI (I)/NADH	*Meiothermus ruber*	50	10	550 ± 60	0.50 ± 0.01	0.9 ± 0.2	^27^
Mt-KARI (I)/NADPH	*Mycobacterium tuberculosis*	37	4	110 ± 4	1.4 ± 0.02	12.73	^13^
Sa-KARI (I)/NADPH	*Staphylococcus aureus*	25	50	285 ± 41	0.23 ± 0.01	0.81	^26^
Re-KARI (I)/NADPH	*Ralstonia eutropha*	30	3	6200	0.115 (0.191)	0.018	^28^
Ec-KARI (II)/NADPH	*Escherichia coli*	37	10	250 ± 30	2.23 ± 0.1	9.02	^8^
Ec-KARI (II)/NADPH	*Escherichia coli*	37	4	500	1.2	2.4	^29^
Os-KARI (II)/NADPH	*Oryza sativa* (rice)	37	4	11.67	1.4	120	^29^
So-KARI (II)/NADPH	*Spinacea oleracea* (spinach)	30	3	10	1.33 (1.4)	133	^30^
Hv-KARI (II)/NADPH	*Hordeum vulgare* (barley)	25	5	11	0.157 (0.16)	14.27	^31^
Ec-KARI (II)/NADPH	*Escherichia coli*	25	10	280 ± 30	2 ± 0.097	7.142 ± 0.689	^9^
Sco1-KARI (I)/NADPH	*Streptomyces coelicolor*	25	10	1600 ± 200	1.1 ± 0.2	0.687 ± 0.043	^9^
Sco2-KARI (I)/NADPH	*Streptomyces coelicolor*	25	10	12000 ± 800	0.24 ± 0.01	0.02 ± 0.0032	^9^
Sli1-KARI (I)/NADPH	*Streptomyces lividans*	25	10	1200 ± 70	0.7 ± 0.59	0.583 ± 0.064	^9^
Sli2-KARI (I)/NADPH	*Streptomyces lividans*	25	10	9300 ± 800	0.28 ± 0.02	0.03 ± 0.0038	^9^
Sam-KARI (I)/NADPH	*Streptomyces ambofaciens*	25	10	2000 ± 150	0.63 ± 0.5	0.315 ± 0.033	^9^
Sav-KARI (I)/NADPH	*Streptomyces avermitilis*	25	10	800 ± 90	0.24 ± 0.2	0.3 ± 0.039	^9^
Sgr-KARI (I)/NADPH	*Streptomyces griseus*	25	10	1200 ± 700	0.42 ± 0.04	0.35 ± 0.037	^9^
Spr-KARI (I)/NADPH	*Streptomyces pristinaespiralis*	25	10	9000 ± 700	3.6 ± 0.3	0.4 ± 0.038	^9^
Svi2-KARI (I)/NADPH	*Streptomyces viridifaciens*	25	10	1800 ± 900	2 ± 0.07	1.111 ± 0.115	^9^
Cgl-KARI (I)/NADPH	*Corynebacterium glutamicum*	25	10	8000 ± 900	3.2 ± 0.4	0.4 ± 0.015	^9^

The value in parentheses is the original value shown in literature and has the units μmol min^−1^ mg^−1^. In this work, enzyme activities were determined in 100 mM potassium phosphate at pH 8.0 with 220 μM NADPH or NADH, 2–10 mM MgSO_4_ and appropriate dilutions of 2*S*-AL in the concentration ranged from 0.0625 to 5.5 mM.

KARI proteins catalyse the second step of the BCAA biosynthesis pathway. This step is the slowest in the pathway; the reported *k*_*cat*_ values for KARIs from different species range from approximately 0.1 to 2.00 sec^−1 ^^8,13,28–31^. Although the *K*_*M*_ values of most KARIs for 2*S*-AL are in the micromolar range, plant KARIs have much lower *K*_*M*_ values (~11 μM)^29–31^ for 2*S*-AL than prokaryotic KARIs. The measured *k*_*cat*_, *K*_*M*_ and *k*_*cat*_/*K*_*M*_ values of Sac-KARI at three different temperatures are all comparable with those of various KARIs, especially those from bacteria. The plant KARIs exhibit much higher 2*S*-AL activity with NADPH than prokaryotic KARIs due to their very low *K*_*M*_ values for this cofactor.

### Mg^2+^ enhances Sac-KARI activity but affects its thermal stability

Mg^2+^ is absolutely required for KARI activity^4,32^. The enzyme binds two Mg^2+^ ions in the active site to bridge the substrate ligand and protein^25^. It has been demonstrated that Mg^2+^ ions not only play a structural role by facilitating the formation of binding sites for the substrate and the cofactor NAD(P)H but that they also have the essential catalytic function^29^. Plant KARI possesses an extremely high affinity for Mg^2+^ (*K*_*M*_ value 5 μM)^30,32^ compared to its bacterial and fungal counterparts, which have much lower Mg^2+^ affinities (*K*_*M*_ values of 420–900 μM)^4,8,23^. The assay buffers used in the enzyme kinetic assays for various KARIs shown in Table 2 in the presence of either cofactor contain Mg^2+^ concentrations ranging from 3 to 10 mM; these concentrations are saturating and exceed the *K*_*M*_ values of most other KARIs for Mg^2+^ by more than tenfold.

In our study, two Mg^2+^ concentrations are used in the kinetic assay. Whereas 2 mM is probably not a saturating Mg^2+^ concentration for Sac-KARI, 10 mM is a saturating concentration for Sac-KARI judging from the *K*_*M*_ value for Mg^2+^ of bacterial KARI^4,8,23^. In our experiments, all *K*_*M*_ values for 2*S*-AL in 10 mM Mg^2+^ were 1.3-2.5-fold lower than those in 2 mM Mg^2+^ under various experimental conditions. However, the 2*S*-AL *k*_*cat*_ values increased only slightly at the higher Mg^2+^ concentration at 25 and 37 °C, with the enzyme showing 1.3-2.8-fold greater catalytic efficiency for 2*S*-AL from 25 to 60 °C. The Mg^2+^ concentration primarily affects substrate binding by Sac-KARI and has only a minor influence on the catalytic rate. The fact that the substrate binding affinity of Sac-KARI increased at increased Mg^2+^ concentration indicates that the presence of sufficient Mg^2+^ ions is crucial for substrate binding to the protein. Interestingly, inconsistent *K*_*M*_ and *k*_*cat*_ values for *E*. *coli* KARI with 2*S*-AL as substrate at 37 °C have been reported in the literature^8,9,29^ (Table 2). The main difference in the reported experimental conditions is the Mg^2+^ concentration. In the presence of 10 mM Mg^2+^, Ec-KARI displayed a *K*_*M*_ value of 250 μM and a *k*_*cat*_ value of 2.231 sec^−1 ^^8^. A comparable *K*_*M*_ value of 280 μM and *k*_*cat*_ value of 2.0 sec^−1^ has also been reported^9^, whereas a higher *K*_*M*_ of 500 μM and a lower *k*_*cat*_ of 1.2 sec^−1^ were seen with 4 mM Mg^2+ ^^29^. The catalytic efficiency of Ec-KARI for 2*S*-AL is thus 3.76-fold greater at the higher Mg^2+^ concentration. This finding is consistent with our observations that the substrate binding and catalytic efficiency of KARI are highly dependent on the Mg^2+^ concentration.

It is worth noting that the Sac-KARI protein becomes unstable and aggregates when incubated at 60 °C in the presence of NAD(P)H and Mg^2+^. Heating the enzyme for several minutes causes an increase in the absorbance of the enzyme solution at 340 nm, particularly at high Mg^2+^ concentrations. Although the secondary structure of Sac-KARI remains intact in the presence of 2 mM Mg^2+^ at 60 °C (Fig. 3C), in the presence of 10 mM Mg^2+^ and 0.22 mM NADPH at 60 °C, the absorbance at 340 nm first decreases with time from 1.1 to 0.59 for ~75 seconds after the addition of 5 mM 2*S*-AL and then increases up to 1.6 in 75 to 150 seconds. This is most likely due to the formation of soluble protein aggregates that cause dynamic light scattering in the wavelength range of 320–400 nm. The extent of Sac-KARI aggregation is highly correlated with Mg^2+^ concentration at high temperature. The protein aggregates slightly in 2 mM Mg^2+^ at 60 °C in the presence of either NADH or NADPH. However, when Sac-KARI is incubated with 10 mM Mg^2+^ and 0.22 mM NADH, a cloudy solution immediately forms upon the addition of 2*S*-AL. For this reason, in our kinetic experiments using NADH as a cofactor at 60 °C, the Mg^2+^ concentration was decreased to 6 mM. When the Mg^2+^ concentration was increased from 2 to 10 mM (6 mM for NADH) at 60 °C, the *k*_*cat*_ values decreased from 0.957 to 0.876 with NADPH and from 0.526 to 0.444 with NADH, probably due to protein aggregation. Nevertheless, the enzyme still appears more active at the higher Mg^2+^ concentration at 60 °C due to the significant decrease in *K*_*M*_ values. The reversed increment in the absorbance at 340 nm with time (due to protein turbidity) does not occur at 25 or 37 °C.

### The activity of Sac-KARI is enhanced at high temperatures

Because Sac-KARI is a thermostable enzyme, we were able to study the temperature dependence of the kinetic parameters for 2*S*-AL by increasing the temperature from 25 to 60 °C (Table 2). The *K*_*M*_ values of KARI for 2*S*-AL with NADH as a cofactor exhibit the same trend as those obtained with NADPH but are higher than those measured with NADPH at all temperatures examined. At 25 °C, Sac-KARI exhibits 1.3-2.5-fold higher *K*_*M*_ for 2*S*-AL with NADH than with NADPH, although the *k*_*cat*_ values are similar. The differences in the *K*_*M*_ and *k*_*cat*_ values in the presence of the two cofactors become more prominent when the temperature is raised. The *K*_*M*_ values decreased 2.0-2.3-fold (2.1-3.3-fold), the *k*_*cat*_ values increased 12.9-16.5-fold (7.2-9.6-fold), and the *k*_*cat*_/*K*_*M*_ values increased 30.0-32.2-fold (14.4-30.5-fold) for 2*S*-AL with NADPH (NADH) when the temperature was increased from 25 to 60 °C. The kinetic behaviour of Sac-KARI as a function of temperature is similar when either NADPH or NADH is used as the cofactor. Our results show that the affinity between 2*S*-AL and Sac-KARI increases with temperature, as shown by the decreasing *K*_*M*_ values, whereas the *k*_*cat*_ values increase over this temperature range. Thus, the overall catalytic efficiency (*k*_*cat*_/*K*_*M*_) also increases significantly with temperature, and temperature affects not only the substrate binding affinity but also the catalytic efficiency of Sac-KARI.

Increased temperature has two major effects on enzymes: the catalytic rate increases, and denaturation also occurs. In contrast to the rate parameters (*V*_*max*_ or *k*_*cat*_), which typically increase as the temperature rises, the temperature-dependent changes in *K*_*M*_ show variations^33–35^. There have been a number of general observations that the *K*_*M*_ values of mesophilic enzymes often increase with temperature^34,35^. However, the thermophilic Sac-KARI displays the opposite trend; its *K*_*M*_ for 2*S*-AL decreases with increasing temperature. The decreasing *K*_*M*_ value for 2*S*-AL with temperature suggests that Sac-KARI undergoes a conformational change that enhances substrate binding affinity at high temperatures.

With NADPH as cofactor, Sac-KARI displays *k*_*cat*_ values that increase exponentially with temperature in a manner consistent with the Arrhenius equation. From this equation, the activation energy (Ea) was determined to be 64.2 and 59.8 kJ/mol in the presence of 2 mM and 10 mM Mg^2+^, respectively (Supplemental Fig. S5). The results indicate that the enzyme faces a lower energy barrier and catalyses the reaction more efficiently at the higher Mg^2+^ concentration. This further demonstrates that Mg^2+^ plays a major role in the substrate binding and catalytic activity of Sac-KARI.

### Sac-KARI is a bi-cofactor-utilizing enzyme for NADPH and NADH

In Table 3, the kinetic parameters of Sac-KARI towards two cofactors NADPH and NADH with saturating concentrations of 2*S*-AL are summarized and compared with those of other KARI variants that possess different cofactor preferences. Sac-KARI has very low *K*_*M*_ values for both NADH and NADPH, but its *K*_*M*_ for NADH (6.0 μM) is ~15-fold higher than its *K*_*M*_ for NADPH (0.4 μM). In contrast, the *k*_*cat*_ values are very similar, 0.056 s^−1^ for NADH and 0.064 s^−1^ for NADPH. The catalytic efficiency (*k*_*cat*_/*K*_*M*_) of Sac-KARI with NADPH is thus ~20-fold higher than that of the enzyme with NADH (185.8 vs. 9.5 mM^−1^ s^−1^). Discrimination between NADPH and NADH occurs mainly at the initial binding step. Nevertheless, although Sac-KARI substantially prefers NADPH as a cofactor, it also displays good activity with NADH as a cofactor and can therefore be classified as an NADH-utilizing enzyme. Thus, our kinetic results using the two cofactors demonstrated that Sac-KARI can use both NADPH and NADH with good activity at low concentration but that the enzyme favours NADPH over NADH by approximately 20-fold, primarily as a result of a decrease in *K*_*M*_.

**Table 3:** Kinetic parameters of KARI variants determined using NADPH or NADH with saturating concentrations of 2*S*-AL as substrate.

	Origin	β2-αB loop	*K*_*M*_ (μM)		*k*_*cat*_ (s^−1^)		*k*_*cat*_/*K*_*M*_ (mM^−1^ s^−1^)		NADH/NADPH ratio of *k*_*cat*_/*K*_*M*_	Cofactor preference	Ref
			NADH	NADPH	NADH	NADPH	NADH	NADPH			
Sac-KARI	*Solfolobus acidocaldarius*	LEREGNS	6.0 ± 0.8	0.4 ± 0.2	0.056 ± 0.001	0.064 ± 0.001	9.5 ± 1.3	185.8 ± 80.5	0.051	NADPH	This work
Ia-KARI	*Ignisphaer aaggregans*	LERQGDS	<1	<1	0.02	0.03	>20	>25	~0.8	Bispecific	^6^
Ms-KARI	*Metallosphaera sedula*	LEREGKS	24	31	0.06	0.07	2.5	2.1	1.2	Bispecific	^6^
Hs-KARI	*Hydrogenobaculum sp*.	LDDKSPH	39	46	0.12	0.12	3.2	2.7	1.2	Bispecific	^6^
Sw-KARI	*Syntrophomonas wolfei*	LRKPFDEASEKE	57	44	0.28	0.22	5.0	5.0	1.0	Bispecific	^6^
Tp-KARI	*Thermacetogenium phaeum*	DIPSSEN	<1	40	0.46	0.25	>460	6.0	74	NADH	^6^
Ua-KARI	Uncultured Archaeon	ETEILGGNKNPS	1.1	38	0.22	0.05	200	1.3	152	NADH	^6^
Af-KARI	*Archaeoglobus fulgidus*	LPEWDKAT	5.0	26	0.1	0.04	20	1.5	13	NADH	^6^
Do-KARI	*Desulfococcus oleovorans*	QLEGDAY	32	n.a.	0.25	n.a.	8.0	n.a.	—	NADH	^6^
Mr-KARI	*Meiothermus ruber*	LRPGSRN	240	20	1.09	0.98	4.6	54.5	0.084	NADPH	^27^
So-KARI	*Spinacea oleracea* (spinach)	LRKGSNS	101	1.7	0.95	1.33	9.4	782.4	0.012	NADPH	^30^
Se-KARI	*Slackia exigua*	LREGSSS	45	1.0	0.41	0.8	9	800	0.011	NADPH	^20^
Ec-KARI	*Escherichia coli*	LRKEAIAEKRAS	1075	41	0.3	3.6	0.3	88	0.003	NADPH	^17^
Ec-KARI^6E6^	*Escherichia coli*	LRKE**S**IAEK**D**A**D**	30	650	2.3	0.20	74	0.40	185	NADH	^17^
Se-KARI^DD^	*Slackia exigua*	LREG**D**S**D**	113	880	0.97	0.10	9	0.11	81.8	NADH	^20^
Se-KARI^DDV^	*Slackia exigua*	LREG**D**S**D**	47	>1000	1.01	0.25	22	0.25	88	NADH	^20^

Ec-KARI^6E6^ mutant: A71S, R76D, S78D, Q110V; Se-KARI^DD^ mutant: S61D, S63D; Se-KARI^DDV^ mutant: S61D, S63D, I95V.

Sac-KARI: The Michaelis-Menten constants for the cofactors were measured with appropriate dilutions of NADPH and NADH (0.01~0.22 mM) in the presence of saturating concentrations of substrate 2*S*-AL (5 mM), 10 mM MgSO_4_ and 100 mM potassium phosphate at pH 8.0. Mr-KARI: the kinetic parameters measured at 50 °C.

In previous studies, bispecific KARIs showed roughly equal catalytic efficiencies in the presence of NADPH and NADH^3^. The activity of Sac-KARI in the presence of either NADPH or NADH is higher than the corresponding activities of all four of the bispecific KARIs listed in Table 3 with the exception of Ia-KARI with NADH. The difference in the catalytic efficiencies of Sac-KARI and these three bispecific KARIs (Ms-, Hs- and Sw-KARI) largely results from differences in *K*_*M*_ and not from differences in *k*_*cat*_. Ia-KARI displayed high catalytic efficiency (*k*_*cat*_/*K*_*M*_ > 20) in the presence of NAD(P)H due to its extremely low (sub-micromolar) *K*_*M*_ for both cofactors. The *K*_*M*_ value of Sac-KARI for NADPH is of the same order of magnitude as that of Ia-KARI, which has the smallest reported *K*_*M*_ for NADPH among KARIs from various organisms.

Regarding the naturally occurring NADH-preferring KARIs, Tp-KARI, Ua-KARI and Af-KARI, the *K*_*M*_ values for NADH of these enzymes (<1, 1.1 and 5 μM) are similar to but smaller than that of Sac-KARI (6.0 μM). However, the *k*_*cat*_ values of these KARIs for NADH are 2-8-fold higher than that of Sac-KARI. Thus, Sac-KARI exhibits comparable or slightly lower NADH binding affinity but much lower activity than these native NADH-preferring KARIs (Tp- and Ua-KARI). Compared with engineered NADH-preferring enzymes, Sac-KARI has stronger NADH binding affinity but a lower NADH *k*_*cat*_ value. Hence, Sac-KARI displays catalytic efficiency similar to that of Se-KARI^DD^, but it is less active than Se-KARI^DDV^ and Ec-IlvC^6E6^.

It is worth noting that spinach KARI^30^ and Se-KARI^20^ have been classified as NADPH-preferring enzymes due to their much higher activities in the presence of NADPH (catalytic efficiency >780) than in the presence of NADH. Spinach KARI and Se-KARI both exhibit higher *K*_*M*_ for NADH but similar or even higher *k*_*cat*_ values than the NADH-utilizing KARIs listed in Table 3. Spinach KARI and Se-KARI also display moderate activity in the presence of NADH (catalytic efficiency values of 9–10) and can thus be classified as NADH-utilizing KARIs. Interestingly, the kinetic properties of Sac-KARI show the same trend in the presence of NADH and NADPH as spinach KARI and Se-KARI, with comparable efficiency toward NADH (lower *K*_*M*_ and *k*_*cat*_). However, Sac-KARI is less active toward NADPH (comparable *K*_*M*_ but much lower *k*_*cat*_). Therefore, spinach KARI and Se-KARI possess higher cofactor specificity for NADPH than Sac-KARI, but these three KARIs are all NADH-utilizing enzymes with moderate activity. In conclusion, Sac-KARI, Se-KARI and spinach KARI all possess 7-residue cofactor binding loops, and the results of kinetic assays reveal that they are all NADPH/NADH-utilizing enzymes that prefer NADPH over NADH.

## Discussion

Previous steady-state kinetic and product-inhibition studies have demonstrated that the kinetic mechanism of KARI activity involves random binding of Mg^2+^ and NAD(P)H followed by binding of the substrate acetohydroxy acid^4,30,36^. In plant KARIs (spinach and rice)^22,24^ and in the prokaryotic class I KARIs (i.e., Ia-KARI and Se-KARI)^6,20^, binding of Mg^2+^ and NADPH draws the domains together, closing the active site through the motion of helix α1 to pre-organize the enzyme for substrate binding. Conversely, the active site of Ec-KARI is closed in the apo form, and cofactor binding opens the inter-domain hinge and allows the substrate to enter^23^. The Sac-KARI crystal structure shows an “open” state with the active site exposed to solvent, similar to the structures of apo-Ia-KARI and Mt-KARI-Mg^2+^ complex (Supplemental Fig. S2). We believe that Sac-KARI shares an induced-fit mechanism in which the active site closes upon cofactor binding that is similar to that observed in the prokaryotic class I KARIs and plant class II KARIs but distinct from that of Ec-KARI. The active site plasticity allows KARI to accommodate promiscuous substrates. Having at least four different native substrates, the substrate promiscuity of KARI is essential for the biosynthesis of the branched-chain amino acids. Moreover, Verdel-Aranda *et al*. have obtained a broad range of the enzyme kinetic parameters for ten KARI homologues from *Streptomyces* and *Corynebacterium*, using eight chemically diverse substrates, including the direct proline precursor pyrroline-5-carboxylate (some kinetic parameters toward 2*S*-AL are listed in Table 2). The results reveal a novel amino acid biosynthesis interlock mediated by enzyme promiscuity^9^. Interestingly, like Sac-KARI in this study, many KARIs are also cofactor promiscuous enzymes that can use either NADH or NADPH as the cofactor^6,27^. Enzyme promiscuity is of significant importance because it can serve as a starting point for the evolution of new function in nature.

Several KARI holo-structures confirm that only the β2-αB loop is involved in interactions with the adenosine 2′-group of NAD(P)H and thus is responsible for the cofactor specificity. Se-KARI and So-KARI each has a 7-residue cofactor binding loop, in which the four binding residues for the adenosine 2′-phosphate are identical (Supplemental Table S1). In the crystal structure of spinach KARI^17,20^, the oxygen atoms of the 2′-phosphate form hydrogen bonds with the side chains of residues R162, S165, and S167. The equivalent residues in Se-KARI are R58, S61 and S63, and these residues also interact directly with the 2′-phosphate of the cofactor (Supplemental Fig. S3)^20^. However, in the crystal structure of the bispecific holo-Ia-KARI, the binding mode for NADP has only one S53 at the end of the loop. The side chain of R49 undergoes the typical packing interaction against the adenine ring and forms a salt bridge with the phosphate of the cofactor^7^. Our Sac-KARI crystal structure contains a sulphate at the cofactor binding site, implying that R49 and S53 are involved in binding the 2′-phosphate of NADPH as in Ia-KARI and Se-KARI (Supplemental Fig. S3). The kinetic data presented in Table 3 show that Sac-KARI, Ia-KARI, Mr-KARI, Se-KARI, and spinach KARI are all NADH-utilizing enzymes but that their catalytic efficiency in the presence of NADH is equal to or lower than that in the presence of NADPH. Interestingly, the KARI enzymes that have similar loop sequences or cofactor binding modes as deduced from their crystal structures also exhibit similar kinetic behaviour and cofactor preferences.

Notably, changes in cofactor specificity could be achieved through amino acid substitutions, insertions, or deletions in the cofactor binding loop resulting from either natural evolution^6,7^ or artificial engineering^17,20,21^. The negatively charged phosphate of NADPH often interacts with positively charged residues, particularly arginine, and with hydrogen-bond-donating residues such as serine. In contrast, in NADH-preferring enzymes, negatively charged residues often serve to repel the NADPH phosphate and accept hydrogen bonds from the ribose 2′ and 3′-OH groups. The last serine residue in the cofactor binding loop is highly conserved, and it is undoubtedly involved in binding both cofactors. This serine interacts with NADPH by forming hydrogen bonds either with the oxygen of the 2′-phosphate, as seen in Se-KARI and spinach KARI, or with both the 3′-OH and the 2′-phosphate, as observed in *Azotobacter vinelandii* KARI (Aa-KARI) and Ia-KARI. In the NADH binding mode, it forms H bonds with the 2′ and 3′-OH groups of the adenosine ribose, as shown in Ua-KARI, a NADH-preferring enzyme^7^. Hence, the serine residue at the end of the loop plays a key role in cofactor binding but does not determine the enzyme’s cofactor specificity.

To date, all native NADH-utilizing KARIs have been found in thermophilic, acidophilic or halophilic microbes that are adapted to extreme conditions^6,7,27^. In this study, we have demonstrated that Sac-KARI is a NADH-utilizing enzyme from the Crenarchaeota that also displays thermophilic and acidophilic characteristics. *Sulfolobus acidocaldarius* is a thermoacidophilic archaeon that was isolated from hot springs where the pH is less than 3 and the temperature ranges from 65–90 °C. Sac-KARI displays 2*S*-AL activity with the lowest *K*_*M*_ value of 60 μM and the highest *k*_*cat*_/*K*_*M*_ value of 14.6 mM^−1^ s^−1^ with NADPH at 60 °C. It shows the lowest *K*_*M*_ values of 91 μM and the highest catalytic efficiency of 4.88 mM^−1^ s^−1^ toward 2*S*-AL with NADH under the same conditions (Table 2). Sac-KARI exhibits moderate activity towards NADH at saturating concentration of 2*S*-AL, which is similar to spinach KARI, Se-KARI and Se-KARI^DD^ with NADH catalytic efficiency values of 9–10 mM^−1^ s^−1^ and is better than most other bispecific KARIs. Also a bi-cofactor-utilizing enzyme that prefers NADPH over NADH, Mr-KARI is thermoactive at 50 °C, but when comparing the enzyme activity towards 2*S*-AL, NADPH and NADH, Sac-KARI performs better than Mr-KARI^27^. However, the catalytic efficiency of Sac-KARI by using NADH is much lower than the real NADH-preferring KARIs^6,20^ (native Tp- and Ua-KARI, *K*_*cat*_/*K*_*M*_: 460 and 200 mM^−1^ s^−1^; engineered Ec-KARI^6E6^, *K*_*cat*_/*K*_*M*_: 74 mM^−1^ s^−1^), which should be better candidates than Sac-KARI in anaerobic fermentation application. In addition, bispecific Ia-KARI seems to be another good choice due to its high thermostability, with half-maximal residual activity (*T*_50_) at 95 °C^6^.

Many enzymes from thermophiles are less active at room temperature but exhibit high activity when the temperature approaches the growth temperature of the organism^26,37,38^. In comparison, Sac-KARI is less active than its mesophilic counterparts at temperatures ranging from 25 to 37 °C. However, the activity of Sac-KARI at 60 °C, which is close to its physiological temperature, is comparable to that of other prokaryotic KARI variants at their growth temperatures. Like most thermophilic enzymes, Sac-KARI is optimally active at temperatures close to the host organism’s optimal growth temperature. Numerous studies have reported that various enzymes from thermophilic and mesophilic organisms have similar activities and catalytic efficiencies under their respective physiological conditions^33,37,38^. It seems difficult to enhance protein stability without deleterious effects on activity; in other words, a protein cannot be both hyperstable and an optimal catalyst concurrently. Nevertheless, enzymes that are active at higher temperatures offer major biotechnological advantages including permitting the use of higher substrate concentrations, making it easier to avoid microbial contamination of reactions, and often yielding higher productivity and lower activity losses during processing^39^. In conclusion, Sac-KARI is one of the rare enzymes that displays high activity at elevated temperatures, a board range of pH tolerance and is able to utilize both NADH and NADPH as co-factors. These properties make it a potential candidate for use in metabolic engineering or industrial applications under anaerobic or harsh conditions.

## Methods

### Gene cloning and protein overexpression and purification

The gene encoding the ilvc1559 (Sac-KARI) was amplified from the *S*. *acidocaldarius* genomic DNA using PCR and cloned into the pET-21a vector using the NdeI and XhoI restriction sites while retaining the His-tag coding region. The plasmid, which was designated pET21a-ilvc, was then transformed into *E*. *coli* BL21 (DE3), and the transformed cells grown at 37 °C in ampicillin-containing LB medium. When the OD_600_ of the culture reached 0.8, Sac-KARI expression was induced by adding 0.5 mM isopropyl β-D-thiogalactopyranoside (IPTG).

After 5 hours of incubation, the cells were harvested by centrifugation and broken by sonication in lysis buffer (buffer A) containing 20 mM Tris-HCl pH 7.5, 50 mM NaCl, 2 mM MgCl_2_ and 5 mM β-mercaptoethanol; the cell debris was then removed by centrifugation. The supernatant was heated to 65 °C for 30 min to remove most of the host proteins. The supernatant was loaded onto a Ni-NTA column (His-Trap F55, GE Healthcare Life Sciences), and the protein was eluted using a gradient of 0–500 mM imidazole in buffer A. Fractions containing His-tagged Sac-KARI were pooled and concentrated using a Centricon membrane (10 K cutoff, GE Healthcare Life Sciences). The protein was further purified and characterized by gel filtration chromatography using a HiLoad 16/600 Superdex 200 pg column (GE Healthcare Life Sciences).

Expression of SeMet-substituted Sac-KARI for phasing purposes was conducted using the Overnight Express™ Autoinduction System 2 (Novagen) containing 125 μg/ml L-methionine and 25 mg/ml SeMet. The protein was purified as described above and concentrated using a Centricon membrane for crystallization. Protein concentrations were determined by measuring the ultraviolet absorbance at 280 nm using an extinction coefficient (ε_280_) of 37,360 M^−1^ cm^−1^ for the Sac-KARI monomer.

### Crystallization, data collection, and structure determination

A Rigaku phoenix/RE robot (Institute of Biological Chemistry, Academia Sinica) was employed in the initial screening for crystallization conditions by mixing 0.5 μl of the Sac-KARI solution with 0.5 μl of each of the various reservoir solutions in sitting-drop vapour-diffusion set-ups at 25 °C. Promising results were obtained by using 7.5–15 mg/ml protein, 15–20% PEG3350, and 0.2 M Li_2_SO_4_ or (NH_4_)_2_SO_4_. Subsequent optimization experiments used 2 μl protein and 2 μl reservoir solution in each drop. The best crystals with highest X-ray diffraction resolution, however, were not obtained by using the native protein but with 10 mg/ml SeMet-containing Sac-KARI and a reservoir solution of 0.2 M Li_2_SO_4_ and 20% PEG 3350.

The X-ray diffraction data were collected at beamlines 13B and 15A of the National Synchrotron Radiation Research Center (NSRRC, Hsinchu, Taiwan) using an ADSC Q315r CCD detector. Ethylene glycol (20%) was used as a cryoprotectant. All data from the native and SeMet crystals, including a multi-wavelength anomalous diffraction (MAD) data set, were processed using HKL2000^40^. All of the Sac-KARI crystals obtained were isomorphous, belonging to space group *P*2_1_2_1_2_1_ and containing one dimer in an asymmetric unit. Because the SeMet crystals diffracted better than the native crystals, one data set collected at a remote wavelength of 1.0 Å was subsequently used in structural analysis. Some of the data collection statistics are given in Table 1.

The crystal structure was solved by molecular replacement (MR) using a dimer from PDB 4XDZ (Ia-KARI from *Ignisphaera aggregans*, with 66% sequence identity to Sac-KARI) as the search model. Two clear solutions related by crystallographic symmetry were obtained using CNS^41^. Initial rigid-body refinement based on either solution yielded an R-value of 0.45 for all data from 30 Å to 1.75 Å. The electron density map, which was calculated with a figure of merit (FOM) of 0.46, clearly showed most side chain differences as well as the locations of many solvent molecules. Manual modification of the model was conducted using COOT^42^. Computational refinement used CNS and PHENIX^43^ with TLS as well as the Refmac5^44^ in CCP4 program suite^45^. Non-crystallographic symmetry (NCS) restraints were included at the early stages but released in the end due to significant deviations between the two monomers. The atomic coordinate and structural factor have been deposited in the Protein Data Bank (PDB ID code 5YEQ). Drawings of the structural models were prepared using PyMOL (The PyMOL Molecular Graphics System, Version 1.7.6.4, Schrodinger, LLC).

### Circular dichroism (CD) spectroscopy

CD spectra were measured using an Aviv Model 410 circular dichroism spectrometer in quartz cuvettes with a 1-mm pathlength. The protein samples were prepared in 50 mM sodium phosphate, pH 8.0 and 2 mM MgSO_4_ at concentrations ranging from 0.15 to 0.3 mg/ml. Temperature-dependent far-UV CD spectra were taken in the wavelength range of 200 to 260 nm with a bandwidth of 1 nm from 25 to 95 °C (25, 65, 75, 85 and 95 °C). Baselines were measured using the buffer and were subtracted from the sample CD signals. The pH-dependent measurements were performed at 25 °C in citric acid/sodium citrate buffer for pH values of 3–5 and in Na_2_HPO_4_/NaH_2_PO_4_ buffer solutions for pH values of 6–8. The thermal melting curve was monitored at 208 nm with an interval of 2 °C and an averaging time of 30 seconds at each temperature from 25 °C to 95 °C. The melting temperature was estimated using a simple two-state model.

### Substrate preparation and quantitative determination

Because 2*S*-acetolactate, a substrate of KARI, was not commercially available, racemic 2-acetolactate was prepared by alkaline hydrolysis of the corresponding esters^4^. Specifically, 1 g of a solution of (±) ethyl-2-acetoxy-2-methylacetoacetate (97% purity, Sigma-Aldrich) was mixed with 4 ml of H_2_O, and 15 ml of 1 M NaOH was slowly added to the mixture at 25 °C for 1 hr. The substrate solution was then adjusted to pH 7.6 with 6 M HCl and stored at −20 °C. In this way, approximately 20.8 ml of 220 mM racemic 2-AL was obtained. The substrate was used directly in activity measurements without further purification. Furthermore, the optical rotations of 2-AL were measured in diluted NaOH solution at 25 °C on a Perkin-Elmer Modle 131 polarimeter. The specific rotation of the substrate solution was ${[\alpha ]}_{D}^{25}$ − 0.15° which is close to zero, confirming that the 2-AL exists as a racemic mixture. The concentration of 2*S*-AL is thus one half of the racemic 2-AL mixture.

The concentration of racemic 2-AL was determined by colorimetrically measuring the amount of its decarboxylation product, acetoin (3-hydroxy-2-butanone). To 1 ml of 2-AL containing solution, 0.240 ml of 6 M H_2_SO_4_ was added, and the mixture was incubated at 80 °C for 10 min. After cooling to 25 °C, the mixture was neutralized by adding 0.235 ml 50% (wt/vol) NaOH to bring the pH of the solution to between 9 and 10. The acetoin in the solution was then quantified by the Westerfield method^46,47^ using creatine/α-naphthol reagents. To each sample, 0.3 ml of 0.5% (wt/vol) creatine and 0.3 ml of 5% (wt/vol) α-naphthol in 2.5 M NaOH were added consecutively, with thorough mixing after each addition. After incubation at 37 °C for 1 hr and centrifugation at 13,000 rpm for 5 min, the absorbance of the samples at 530 nm was measured. A standard curve was established using acetoin solutions prepared from crystalline powder (Alfa Aesar) in the concentration range of 2–10 μg/ml.

### Kinetic analyses

For measurement of the enzymatic activity of Sac-KARI, an assay buffer containing 100 mM sodium phosphate, pH 8.0 and 2 mM MgSO_4_ was used in the gel filtration step of purification. The concentrated enzyme could be stored at −20 °C for several months without activity loss by including 25% glycerol in the storage buffer. To determine the kinetic parameters of the enzyme for 2*S*-AL, Sac-KARI (1.0 μM monomer) was pre-incubated with Mg^2+^ (2–10 mM) and NAD(P)H (0.22 mM) in the assay buffer for 5 min at the specified temperature. The reaction was then initiated by adding 2*S*-AL to a concentration of 0.0625–5.5 mM, bringing the final volume of the assay to 1 ml, and monitored by measuring the decrease in absorbance at 340 nm (ε = 6220 M^−1^ cm^−1^ for NAD(P)H) at 25, 37 or 60 °C using a V-630 spectrophotometer (JASCO International Co.). Quartz cuvettes were used in all experiments due to their relatively rapid temperature equilibration and heat-retaining capacity. To obtain more precise initial velocities, only data obtained before 15% of the NAD(P)H present in the reaction had been consumed were used in calculating the linear rate of each reaction. Due to the possibility of protein aggregation, measurements of initial velocity at 60 °C used only data obtained before 10% of the NAD(P)H present in the reaction had been consumed. The substrate conversion rate as a function of substrate concentration was fitted by non-linear regression (OriginLab 8.0, OriginLab Corporation, USA) to the Michaelis–Menten Equation (1), where V_max_ (=k_cat_[E]_total_) represents the maximum velocity, K_M_ is the Michaelis constant, S is the substrate concentration and V is the initial velocity.

1 $${\rm{V}}=\frac{{V}_{max}\cdot S}{{K}_{M}+S}$$

The kinetic parameters of Sac-KARI for each cofactor were determined in assay buffer containing 10 mM MgSO_4_ and a saturating concentration of 2*S*-AL (5 mM) at 25 °C, with appropriate dilutions of NADPH and NADH (0.0068 mM to 0.22 mM). All measurements were performed in triplicate. The values shown in Tables 2 and 3 represent the average of three independent measurements.

### Data availability

All data generated or analysed during this study are included in this published article (and its Supplementary Information files).

## Electronic supplementary material

Supplementary information
