# Supplementary material for: NADH/NADPH bi-cofactor-utilizing and thermoactive ketol-acid reductoisomerase from Sulfolobus acidocaldarius
Source: Sci Rep. 2018 May 8;8:7176. doi: 10.1038/s41598-018-25361-4 (PMC5940873; doi:10.1038/s41598-018-25361-4)
Supplement: Supplementary file 1 — Supplementary information [file 41598_2018_25361_MOESM1_ESM.pdf]

NADH/NADPH bi-cofactor-utilizing and thermoactive ketol-acid reductoisomerase from *Sulfolobus acidocaldarius*

Chin-Yu Chen[1,+,*], Tzu-Ping Ko[2,+], Kuan-Fu Lin[1], Bo-Lin Lin[3], Chun-Hsiang Huang[4], Cheng-Hung Chiang[4] and Jia-Cherng Horng[5]

[1]Department of Life Sciences, National Central University, Taoyuan 32001, Taiwan

[2]Institute of Biological Chemistry, Academia Sinica, Taipei 11574, Taiwan

[3]Research Center for Applied Sciences, Academia Sinica, Taipei 11574, Taiwan

[4] Protein Diffraction Group, Experimental Facility Division, National Synchrotron Radiation Research Center, Hsinchu 30077, Taiwan

[5]Department of Chemistry, National Tsing-Hua University, Hsinchu 30013, Taiwan

[+]These authors contributed equally to this work.

Corresponding author:

Chin-Yu Chen, Ph.D.

Department of Life Sciences, National Central University,

300 Zhongda Road, Zhongli District, Taoyuan City 32001, Taiwan

E-mail: chinyuchen@cc.ncu.edu.tw

chinyuchen33@gmail.com

Supplementary information

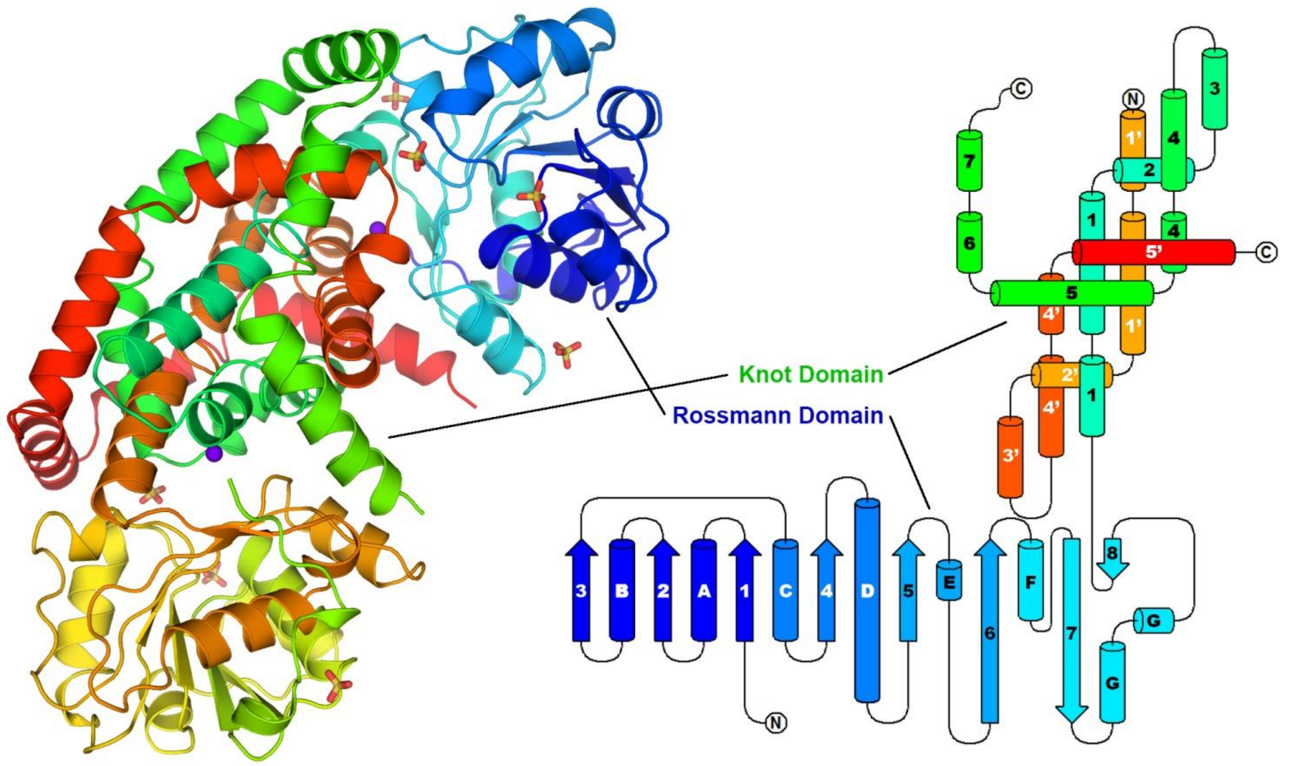

Supplementary Figure S1. Overall structure and topology of Sac-KARI. On the left is a ribbon model of the dimer, coloured from the N to the C termini in blue to green for one monomer and in yellow to red for the other. On the right is a schematic diagram in which the arrows and cylinders represent β-strands and α-helices. Only the knot domain of the second monomer is included here. The figure was constructed using TopDraw.

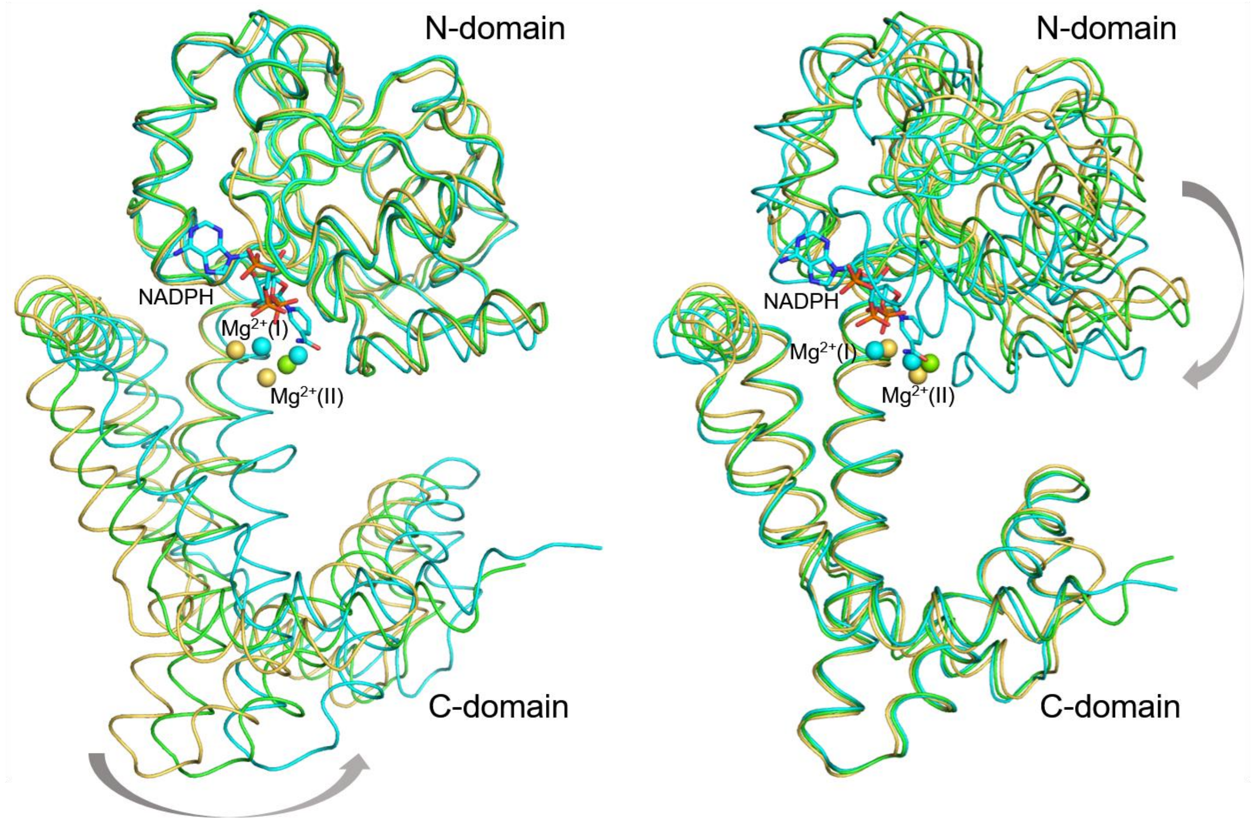

Supplementary Figure S2. Comparison of the conformation of the individual subunit structures for the class I KARIs. Left: N-terminal domain alignment. Right: C-terminal domain alignment. Sac-KARI (PDB code: 5YEQ, one Mg^{2+} in active site) in green, Mt-KARI-Mg^{2+} complex (PDB code: 4YPO, two Mg^{2+} in active site) in yellow and Ia-KARI-Mg^{2+}-NADPH-IpOHA complex in cyan (PDB code: 4XDZ). When comparing the Sac-KARI structure with Mt-KARI and holo-Ia-KARI complexes for local alignment of amino acids, the major difference is seen in the N-domain, which is rotated by a different angle relative to the C-domain and vice versa. When the KARIs are superimposed by using the N-domain, the C-domain rotates towards the N-domain in a progressive way from the Mt-KARI-Mg^{2+} complex to the Ia-KARI-Mg^{2+}-NADPH-IpOHA complex. The C-domain is pulled closer to the N-domain upon the binding of NADPH and IpOHA. Similarly, when the C-domain is used in the alignment, the N-domain of each KARI moves towards the C-domain to a varied extent. The N-domain of the Ia-KARI-Mg^{2+}-NADPH-IpOHA structure is moved the most when compared with Mt-KARI-Mg^{2+}, whereas Sac-KARI shows an intermediate conformation that is closer to Mt-KARI-Mg^{2+}. In addition, a comparison of the Mt-KARI-Mg^{2+} structure with that of Mg^{2+}-free Pa-KARI suggests that

no movement of the N-domain relative to the C-domain occurs upon magnesium binding. Clearly, there is no significant domain movement when magnesium ions bind to class I KARIs. The major consequence of domain movement is that binding of NADPH and substrate (or inhibitor) leads to a reduction of solvent accessibility to the active site. Thus, the active site is more exposed to the solvent in the Mt-KARI-Mg^{2+} complex, less exposed to the solvent in the Sac-KARI, and almost completely closed in the Ia-KARI-Mg^{2+}-NADPH-IpOHA complex.

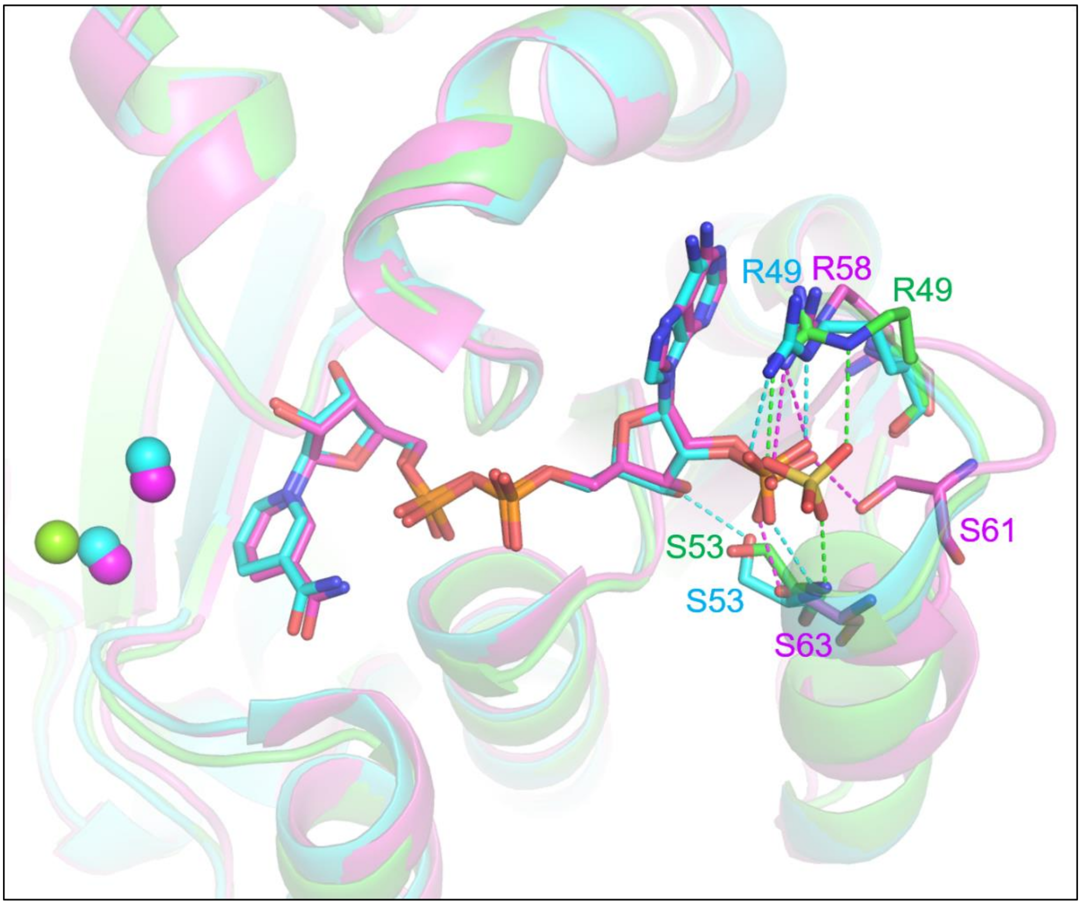

Supplementary Figure S3. Structural alignment of the NAD(P)H-binding pocket of Sac-KARI (PDB code: 5YEQ, green) with Ia-KARI (PDB code: 4XDZ, cyan) and Se-KARI (PDB code: 4KQW, magenta). Sac-KARI binds sulphate while Ia-KARI/Se-KARI binds NADPH. The bound NADPH and sulphate are shown as stick; the bound magnesium ions are shown as spheres; the hydrogen bonds are shown as dashed lines. Despite the difference in the conformations of β2-αB loop for these enzymes, it can be seen that one sulphate on the right is bound to an identical site, as is the 2'-phosphate of NADPH. Residues R49, and S53 in Sac-KARI appear to interact with the 2'-phosphate group of the bound NADPH as in Ia-KARI and Se-KARI. The magnesium ion in Sac-KARI (green sphere on the left) is also close to one of the two magnesium ions in Ia-KARI (cyan) and in Se-KARI (magenta).

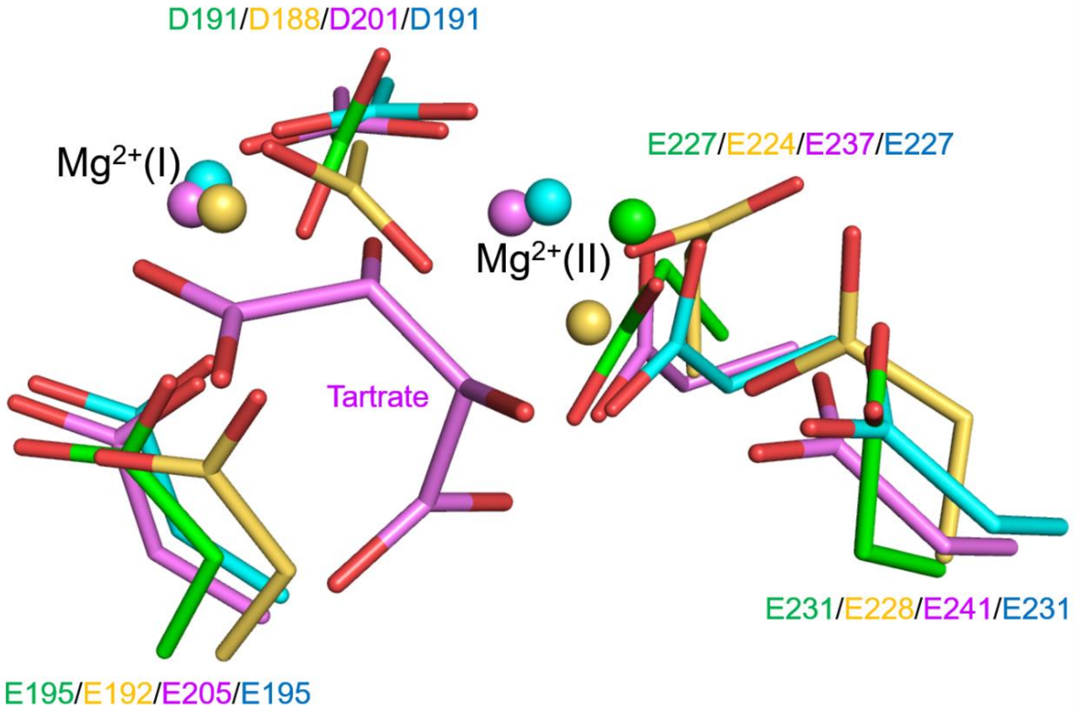

Supplementary Figure S4. Comparison of metal binding sites in class I KARI structures bound to different ligands. Superimposition of metal binding sites in Sac-KARI (green), Mt-KARI-Mg^{2+} complex (yellow), Ia-KARI-Mg^{2+}-NADPH-IpOHA complex (cyan) and Se-KARI-Mg^{2+}-NADPH-tartrate (magenta). Most KARI structures show two Mg^{2+} located within the active site. They are referred to as Mg (I) and Mg (II). They play a major role in substrate (inhibitor) binding to the protein. D191 plays a particular important role by bridging both Mg^{2+}. In Sac-KARI structure, only Mg (II) is observed in the active site. Across the four structures, the location of Mg (I) changes by up to 1.0 Å , while the position of Mg (II) can vary by up to 1.8 Å . Thus, these results indicate that Mg^{2+} locations are variable which depend on the type of ligand present or on the state of catalysis. The reduction of the metal-metal distance from 4.7 Å in the Mt KARI-Mg^{2+} complex to 3.5 Å in the Se-KARI-Mg^{2+}-NADPH-tartrate complex is due to the movement of the Mg (II) ion. Thus, Mg (I) appears to adopt a fixed position in the active site, while Mg (II) moves closer to Mg (II) upon the binding of the substrate (or inhibitor).

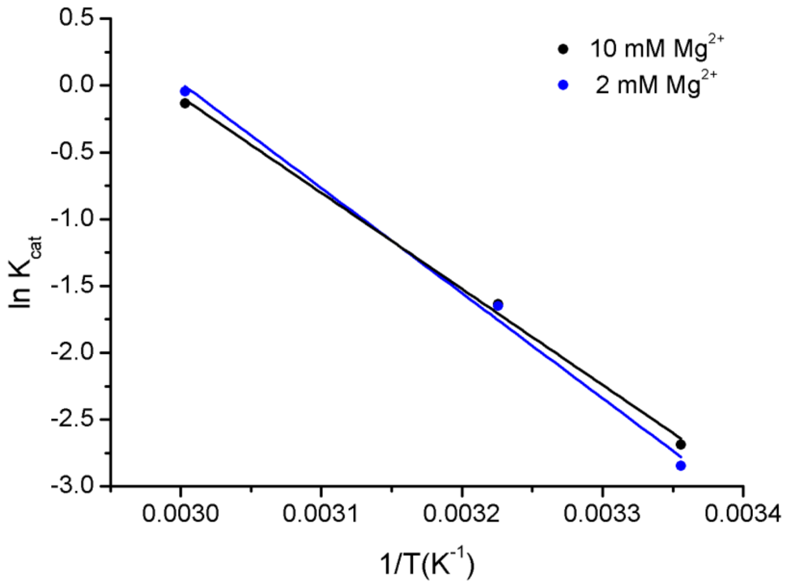

Supplementary Figure S5. Arrhenius plots of Sac-KARI catalysis of 2-AL in the presence of NADPH. By plotting ln k_{cat} versus 1/T, the activation energy for catalysis of 2-AL by Sac-KARI using NADPH was determined to be 59.8 and 64.2 kJ/mol in the presence of 10 mM and 2 mM Mg^{2+}, respectively. Each point represents the average of three measurements; the deviation from the mean was found to be less than the size of the point representing the mean on this plot. In contrast, with NADH as cofactor, the enzyme displays a non-exponential increment in k_{cat} as the temperature is increased from 25 to 60 °C. We measure the Sac-KARI activity in the lower Mg^{2+} concentration (6 mM, instead of 10 mM) at 60 °C due to the protein precipitation. The k_{cat} was obtained not in the same experimental conditions so the activation energy cannot be calculated by the Arrhenius plot. It is necessary to obtain additional values of k_{cat} at temperatures below 60 °C to determine the activation energy of Sac-KARI in the presence of NADH.

Supplementary Table S1. Sequence alignment of cofactor binding loop. Structure-guided alignment of the β2αB-loops (cofactor binding loop) of the KARIs discussed in the present paper. PDB ID, loop length, class of KARI, kingdom of origin and cofactor preference are also given for each enzyme. The residues that contact the 2'-phosphate of NADPH or the 2'-OH of NADH are highlighted in red.

PDB	Source	β2	Loop	αB	Length	Class	Kingdom	Cofactor
**5YEQ**	*S. acidocaldarius*	VSVG	LE--R-------EG-NS	WKQA	7	I	Archaea	NAD(P)H
**4YPO**	*M. tuberculosis*	VRVG	L---K-------QGSRS	RPKV	7	I	Bacteria	NADPH
**1NP3**	*P. aeruginosa*	VTVG	L---R-------SGSAT	VAKA	7	I	Bacteria	NADPH
**4XIY**	*A. vinelandii*	VYVG	L---R-------AGSAS	VAKA	7	I	Bacteria	NADPH
**4KQW**	*S. exigua*	VRVG	L---R-------EGSSS	WKTA	7	I	Bacteria	NADPH
**4XDZ**	*I. aggregans*	VVVG	LE--R-------QG-DS	WRRA	7	I	Archaea	NAD(P)H
**4TSK**	*A. acidocaldarius*	VVIG	L---R-------PG-SS	WAKA	6	I	Bacteria	NADPH
**4XDY**	*Uncultured Archaeon*	VVIV	ETEILGG-----NKNPS	WEKA	12	I	Archaea	NADH
**3ULK**	*E. coli*	ISYA	L---R--KEAIAEKRAS	WRKA	12	II	Bacteria	NADPH
**3FR8**	*O. sativa*	VKIG	L---R-------KGSKS	FDEA	7	II	Eukaryota	NADPH
**1QMG**	*S. oleracea*	VKIG	L---R-------KGSNS	FAEA	7	II	Eukaryota	NADPH
